# The Role of Compensatory Mutations in the Emergence of Drug Resistance

**DOI:** 10.1371/journal.pcbi.0020137

**Published:** 2006-10-13

**Authors:** Andreas Handel, Roland R Regoes, Rustom Antia

**Affiliations:** Department of Biology, Emory University, Atlanta, Georgia, United States of America; The Pennsylvania State University, United States of America

## Abstract

Pathogens that evolve resistance to drugs usually have reduced fitness. However, mutations that largely compensate for this reduction in fitness often arise. We investigate how these compensatory mutations affect population-wide resistance emergence as a function of drug treatment. Using a model of gonorrhea transmission dynamics, we obtain generally applicable, qualitative results that show how compensatory mutations lead to more likely and faster resistance emergence. We further show that resistance emergence depends on the level of drug use in a strongly nonlinear fashion. We also discuss what data need to be obtained to allow future quantitative predictions of resistance emergence.

## Synopsis

Pathogens that evolve resistance to drugs pose a serious public health problem. Acquisition of drug resistance usually leads to a pathogen which in the absence of the drug is less fit than the nonresistant pathogen. However, the resistant pathogen can undergo additional mutations that compensate for the fitness cost involved with acquisition of resistance. This can result in a drug-resistant pathogen with fitness comparable to that of the nonresistant pathogen. Handel, Regoes, and Antia analyzed a mathematical model to explain how this process of compensatory mutations influences the probability of and time to resistance emergence on a population level. The authors found that with compensatory mutations, resistance emergence is faster and more likely. Their study also shows how small changes in levels of treatment can lead to large changes in the time to resistance emergence. They further discuss how our ability to accurately predict resistance emergence will depend on improved estimates of the parameters governing the processes of resistance acquisition and compensatory mutations. The study suggests that compensatory mutations can play an important role in the evolution and spread of resistant pathogens through a population.

## Introduction

Drug-resistant pathogens are found at an alarming rate and few replacement drugs are in sight, suggesting a rapidly increasing public health problem [1–6]. There exists broad consensus that prudent use of antibiotics and antivirals is of critical importance to stop or at least reduce emergence of drug resistance.

### Theoretical between-Host Studies of Drug Resistance

A number of theoretical between-host studies have provided some insights into prudent drug use. Both population-genetic [7–9] as well as epidemiological [10,11] studies analyzed the relationship between drug use and levels of resistance for the community at large. Further studies focused on specific scenarios, such as hospital settings [12,13], the interaction between hospitals and the community [14], and influences of animal reservoirs [15]. Other studies investigated drug resistance for specific pathogens, such as malaria [16], HIV [17,18], HSV [19,20], multidrug-resistant tuberculosis [21,22], and influenza [23,24]. Results obtained from these and other studies have led to an improved understanding of the relation between drug use and resistance emergence. In these studies, it is assumed that in the absence of drugs, the resistant pathogen strain is less fit than the sensitive strain. Drug treatment reduces the fitness of the sensitive strain, which leads to potential resistance emergence. What is not considered is the ability of the resistant pathogen to regain some or most of its fitness through compensatory mutations.

### Experimental within-Host Studies of Compensatory Mutations

Compensatory mutations have been found to play an important role in the evolution of pathogens [25–27]. In vitro studies of streptomycin-resistant Escherichia coli found that the cost associated with resistance was quickly reduced by compensatory mutations, and these compensatory mutations can occur in both the presence and absence of the drug [28–30]. Subsequent studies found that compensatory mutations were also important in vivo. Studies passaging drug-resistant Salmonella typhimurium through mice led to the emergence of strains with increased fitness that retained resistance [31,32]. Further studies suggested that compensatory mutations might play a role for the evolution of resistance in Mycobacterium tuberculosis [33,34], Streptococcus pneumoniae [35], Staphylococcus aureus [36], and the yeast Candida albicans [37]. These and other studies suggest that compensatory mutations play a potentially important role in the evolution of resistance.

### Combining Compensatory Mutations and Epidemiological Models

Despite the importance of compensatory mutations for the process of resistance emergence, we are aware of only one population-genetic study investigating the effect of compensatory mutations on the interplay between drug (pesticide) use and resistance [38]. Epidemiological models that include compensatory mutations currently do not exist. To our knowledge, our study is the first one to include compensatory mutations into a between-host epidemiological model of resistance emergence.

Ideally, we would like to study a system for which we can obtain quantitative predictions. Unfortunately, despite the existing studies on compensatory mutations, no host–pathogen system currently exists—as far as we are aware—for which such quantitative predictions could be made. There is simply not enough data available to allow estimation of all the parameters that are needed to quantitatively describe the process of population-wide resistance emergence.

Currently missing are estimates for the between-host fitness levels of resistant mutants. In epidemiological models, the between-host fitness of a pathogen can be defined through the basic reproductive number *R*_0_, the number of secondary infections caused by one infected host introduced into a fully susceptible population [39,40]. In contrast, within-host studies such as compensatory mutation studies measure fitness through in vitro or in vivo growth rates. While a connection can be made between a pathogen's within-host dynamics and its ability to transmit and infect hosts [41], it is currently not known how within-host fitness measured by pathogen growth rates translate into between-host fitness measured by *R*_0_.

Also unknown are the rates at which hosts infected with one pathogen strain “convert” to hosts infected with another strain. Each pathogen has a certain probability of undergoing mutations. Provided that a mutation confers a competitive advantage, the mutant can outgrow the other pathogens within an infected host. Once the mutant strain dominates, one can consider the infected host as having “converted.” (As we will discuss at the end of this article, assuming that a host is completely dominated by only one strain at a time is probably an oversimplification.) The rates of conversion depend on both the mutation rates and the dynamics of the different strains within a given host. A recent study provides estimates for the probability that a host commensally infected with Helicobacter pylori is taken over by a newly arising mutant [42]. However, the results only apply under very restricted conditions. While estimates of compensatory mutation rates exist, for instance for S. typhimurium [43], no clear connection has been established between these mutation rates and the rates at which a host is converted from being infected with one strain to being infected with another strain.

Last, it is also not known what routes of compensatory mutation a pathogen can undergo in a clinically relevant in vivo setting. One study showed that S. typhimurium can follow different routes of compensatory mutations in vitro and in vivo [32]. In principle, these routes could be a single linear pathway or there could be multiple routes with multiple possibilities for compensatory mutations to increase fitness.

The current lack of data for these three quantities—between-host fitness levels, rates of conversion, and routes of compensatory mutations—unfortunately limits the predictive power of any model. Nevertheless, one can gain important *qualitative* insights into how compensatory mutations influence the dynamics of resistance emergence. This is the main point of our current study.

In the absence of good estimates for all the necessary parameters, we consider a pathogen for which most parameters are well-defined, and then we investigate how changes in the unknown parameters influence the results. To that end, we study a between-host model for gonorrhea transmission dynamics. Many of the parameters defining the transmission dynamics of gonorrhea infections are rather well-known [44–47]. This allows us to study how the unknown parameters related to compensatory mutations affect the emergence of resistance. Additionally, many strains of *Neisseria gonorrhoeae,* the pathogen causing gonorrhea, have been found to be resistant to antibiotics such as penicillins, tetracyclines, and quinolones [48,49]. While the pathogen is still mostly susceptible to third-generation cephalosporins, it is likely to be just a matter of time before resistance reaches levels that render this class of drugs ineffective. Therefore, gonorrhea is a pathogen for which resistance studies are needed to help inform public health policy.

## Materials and Methods

The dynamics of gonorrhea transmission has been analyzed in detail [44–47]. One of the findings from these studies is the concept of core groups [44]. Core groups are broadly defined as subsets of the population in which the disease can persist, even if it cannot persist in the rest of the population. For gonorrhea, core groups consist of sexually active high-risk individuals. Since core groups are most likely the “breeding ground” for resistance, we focus on such a group.

### The Model

We include four different pathogen strains in our model, namely a drug-sensitive strain, a resistant mutant, and two further resistant mutants that have undergone compensatory mutations and thereby regained some of the fitness that was lost in the resistance mutation. The model is given by Equation 1, a flow diagram of the model is shown in Figure 1, and Table 1 summarizes the variables and parameters of the system.

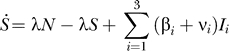


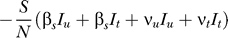


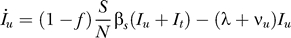


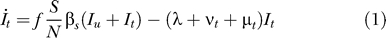


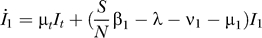


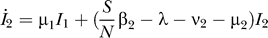


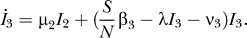



**Figure 1:**
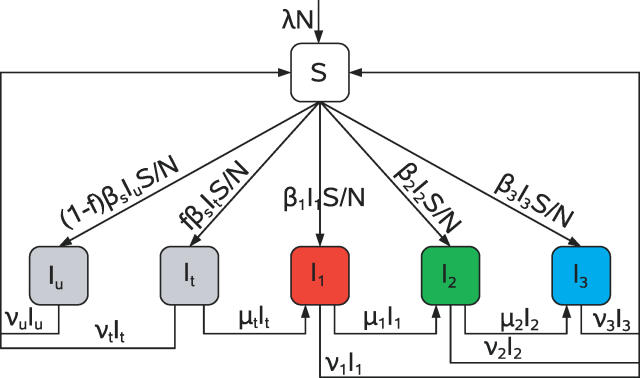
Flow Diagram of the Compartmental Model Describing Gonorrhea Transmission within a Homogenous Core Group Not shown are the flows out of each compartment at rate *λ*. Table 1 summarizes the variables and parameters. A detailed explanation of the model is given in the text.

**Table 1:**
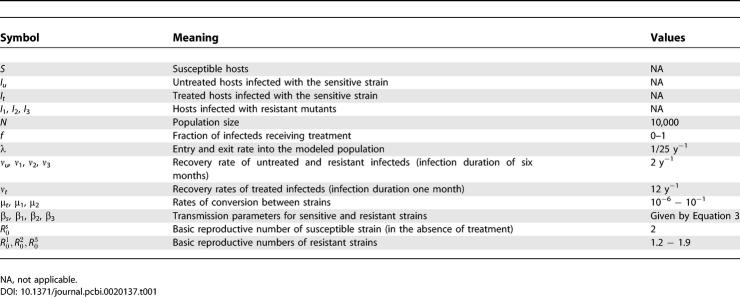
Variables and Parameters of the Model

The details of the model are as follows. We consider a homogeneous core group of sexually highly active people 15–39 years of age. New susceptible individuals *S* enter the group by turning 15 years old and becoming sexually active, and leave the group by turning 40, which can be seen as a proxy for becoming sexually less active. This gives an entry and exit rate of *λ* = 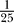 *y* ^−1^. Gonorrhea does not cause significant mortality, and the general death rate is low in persons aged 15–39, therefore we ignore deaths.

Susceptible individuals can become infected through sexual interactions with infected persons at a rate determined by the transmission parameters for the different pathogen strains *β_s_*, *β*_1_, *β*_2_, *β*_3_. We assume that there are no differences in transmission between treated and untreated individuals; treatment only reduces the duration of infection. We specify the transmission parameters through the between-host fitness as measured by the basic reproductive number *R*_0_ (Equation 3). We choose the basic reproductive number for the sensitive strain to be 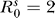 , corresponding to a 50% prevalence in the absence of treatment. Such a high prevalence might occur in certain core groups; for instance, among commercial sex workers. Fitness levels for the resistant strains are chosen at intermediate values such that 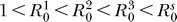 . As mentioned earlier, values for the between-host fitness levels are not known. In a recent study of M. tuberculosis isolated from patients, relative within-host fitness levels between 50% and 100% were reported [34]. While keeping in mind the difficulties of comparing within-host fitness measured by pathogen growth rates and between-host fitness measured by *R*_0_, we use these within-host values as a guideline and choose the between-host fitness for the different resistant strains to be between 60% and 95% that of the sensitive strain.

Some infecteds, *I_u_,* do not receive treatment and remain infectious for six months [44,45]. A fraction *f* of infecteds, *I_t_,* receive treatment and clear the infection after one month. Since N. gonorrhoeae undergoes constant antigenic changes, hosts that clear the infection do not become protected against reinfection and therefore return to the susceptible pool. Instead of recovering, some treated hosts evolve resistance at rate *μ_t_* and convert to *I*_1_. Since we assume that treatment has no effect on hosts infected with the resistant strains, the duration of infection is the same as those of the untreated hosts. The first and second resistant strains can evolve further to convert to the second and third resistant strains at rates *μ*_1_ and *μ*_2_, respectively. As mentioned above, clinically relevant routes of compensatory mutation are not known for any pathogen. Here we choose the simple linear pathway *I*_1_ → *I*_2_ → *I*_3_, with each mutation leading to a strain with increased between-host fitness. Also not known are the rates at which hosts can convert from being dominated by one strain to being dominated by another strain. While it was found in experiments that compensatory mutations are more frequent than mutations resulting in resistance [30], the fitness advantage of a resistance mutation in the presence of drug is likely larger than that of a compensatory mutation. It is not clear how these two effects (and potentially others) influence the overall rates of conversion. We therefore assume for most of our study that all conversion rates are equal, i.e., *μ_t_* = *μ*_1_ = *μ*_2_. We additionally choose values for the conversion rates between 10^−6^ and 10^−1^. We investigate how changes in the conversion rates affect the results.

Figure 2 summarizes the basic dynamics of resistance emergence for our model. The bars indicate the between-host fitness levels as measured by *R*_0_ for the sensitive and resistant pathogen strains. All strains are assumed to have a fitness of *R*_0_ > 1. The sensitive strain can mutate to a resistant strain with strongly reduced fitness. Due to competition between the strains, a given resistant strain can only invade if its fitness is larger than that of the sensitive strain. Therefore, for all but the highest levels of treatment, once the resistant strain is created, it is outcompeted and goes extinct. However, while stuttering to extinction, the resistant strain can undergo further compensatory mutations and thereby increase in fitness. Once the level of fitness is above that of the sensitive strain, the resistant strain can emerge. The level of drug use determines which of the resistant strains can invade.

**Figure 2:**
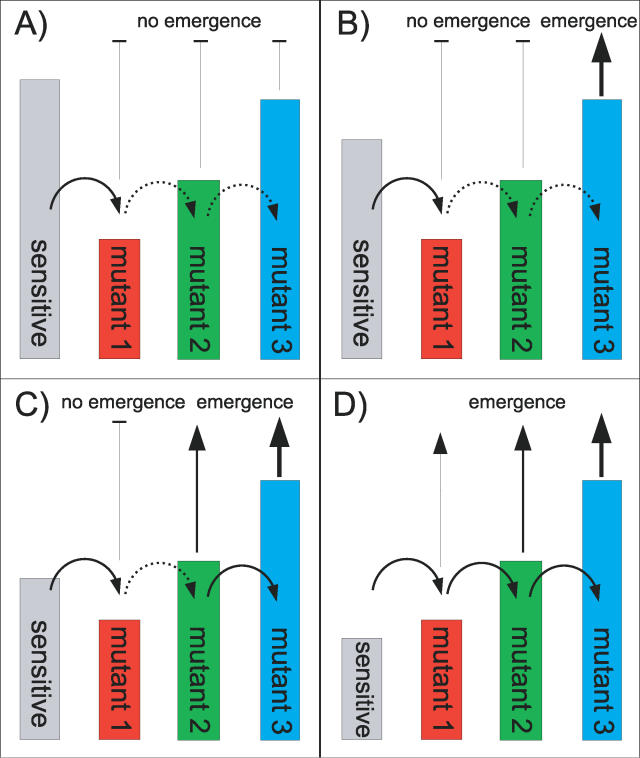
The Process of Resistance Emergence The bars indicate between-host fitness levels of the different strains. Solid curved arrows show conversion events that occur frequently due to large or expanding source populations. Dashed arrows show conversion events that occur infrequently due to small source populations. (A) Without treatment, all resistant strains are less fit than the sensitive strain. Therefore, resistance emergence is not possible. (B) Treatment of a small fraction of the population reduces fitness of the sensitive strain enough to allow for emergence of the fittest resistant strain. For that to happen, one frequent and two rare conversions need to occur. (C) Further increase in treatment level allows both the second and third resistant strains to emerge. For the second resistant strain to emerge, one frequent and one rare conversion need to occur. Subsequently, the third resistant strain is rapidly generated and will outcompete all other strains. (D) Treatment of a large fraction of the population results in all conversion events being frequent and in rapid emergence of resistance.

### Analysis of the Model

Gonorrhea is thought to persist in the population in an endemic state [44], which can be well described by the steady state values of the mathematical model. In the absence of resistant strains, the steady state values are found to be

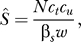


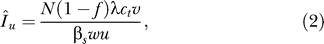


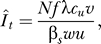
where we introduced the abbreviations *c_u_* = *λ* + *ν_u_*, *c_t_* = *λ* + *ν_t_* + *μ_t_*, *u* = *c_t_λ* + *c_u_fλ* + *c_u_fμ_t_* – *c_t_fλ*, *v* = *c_t_β_s_* − *c_t_c_u_* − *β_s_c_t_f* + *β_s_c_u_f*, and *w* = *c_t_* – *c_t_f* + *fc_u_*. The between-host fitness levels of the different strains are given by their basic reproductive numbers

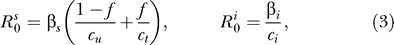
for the sensitive strain and the *i* = 1,2,3 resistant strains, respectively. We defined *c*_1_ = *λ* + *ν*_1_ + *μ*_1_ and *c*_2_ = *λ* + *ν*_2_ + μ_2_. The relative fitness of the resistant strains compared with the sensitive strain can be defined as

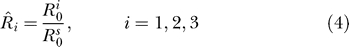



The dynamics of the resistant strains can be computed if we assume that during the process of resistance emergence, the endemic steady state does not change. This is a good approximation if the resistant strains are at a low level, which corresponds to the initial phase of resistance emergence that we consider here. If we assume that the system is at steady state and that initially no resistant pathogens are present, we find the explicit expressions for *I*_1_(*t*), *I*_2_(*t*), and *I*_3_(*t*) to be

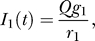


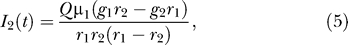


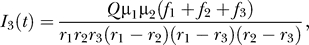
with the abbreviations 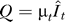 , 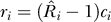 , 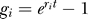 , 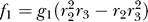 , 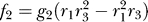 , and 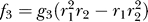 . *Q* is the rate at which resistant mutants are introduced. If the fitness of resistant strain *i* at treatment level *f* is below that of the sensitive strain, the values of 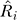 are smaller than one, resulting in *r_i_* < 0 and equilibrium values for the populations *I_i_,* given by

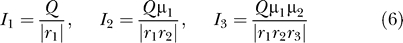
These equilibrium values correspond to a dynamic balance between influx of resistant mutants *Q* and extinction due to being outcompeted by the sensitive strain. If the fitness of a given resistant strain at treatment level *f* exceeds that of the sensitive strain, the corresponding 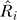 is greater than one and exponential growth occurs. In this case, the time *T_i_* at which the population *I_i_* has reached a certain level *L* is given by the implicit equation

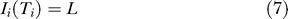
One can obtain an explicit expression for *T*_1_. For the times *T*_2_ and *T*_3_, the equation needs to be solved numerically once the level of resistance *L* has been specified.

We also study a stochastic version of the model (Equation 1). In the stochastic version of the model, the rate parameters of the deterministic model become probabilities that a given event happens. We can obtain the probability of and time-to-resistance emergence using a branching process formalism [50–53]. In our model, the relative fitness 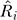 associated with each resistant strain represents the mean number of secondary infections. Since 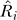 is given in units of generation time, we need to rescale the conversion rates accordingly by defining 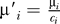 . Following the formalism described in detail in [53], we find that for our three-strain model, the probability of emergence for a single resistance introduction is

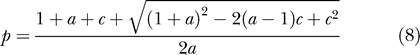
with 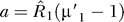 and 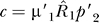 , and the probability *p′_2_* is again given by Equation 8 with 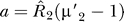 and 
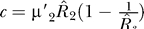
 . Combining the result for *p* with the number of introductions *Q* leads to the probability of emergence in *τ* years

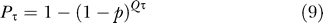



## Results

### The Rate of Resistance Introductions

We consider a scenario where the infection is at an endemic equilibrium, which is believed to be the case for gonorrhea [44]. In the absence of any resistant strains, we obtain from the deterministic model the steady state values 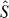 *, Î_u_,* and *Î_t_* (Equation 2). At steady state, hosts infected with the resistant strain are introduced at a rate *Q* = *u_t_ Î_t_*. Figure 3 shows the number of introductions per year as a function of *f*. As *f* increases, the fraction of treated hosts increases. At the same time, treatment reduces the total number of infected hosts. Competition between these two effects determines the shape of *Î_t_,* and thereby *Q,* as a function of *f*. In general, the number of introductions is zero at *f* = 0 and at some level *f* ^*^, at which treatment drives the pathogen to extinction. The maximum number of introductions is found for some intermediate value of *f* and depends on the parameters of the system.

**Figure 3:**
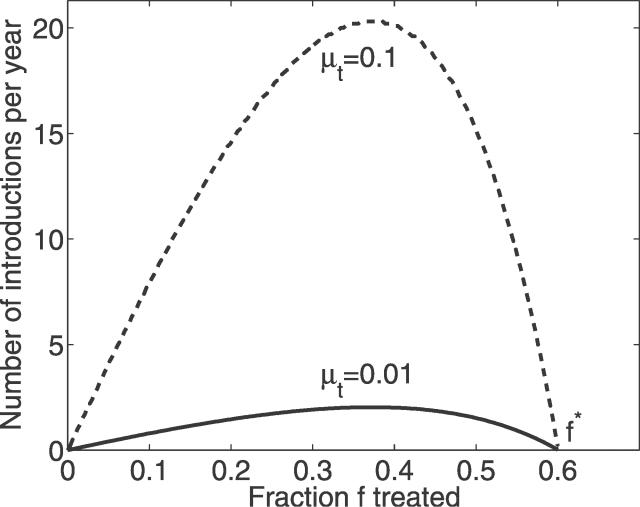
Number of Introductions *Q* = *μ_t_ Î_t_* per Year Parameter choices are described in the text and in Table 1. Note that above a certain level of treatment, indicated by f*, the basic reproductive number for the sensitive strain is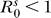 , which makes the endemic steady state unsustainable and leads to disease extinction. In this study, we only consider the endemic situation with 0 ≤ *f* < f*.

### Time to Emergence for the Deterministic Model

A first guess might suggest that one should try to minimize the number of introductions *Q* to maximize the time until resistance emerges. However, the whole process of resistance emergence also depends on the competition of the sensitive and resistant strains. If the relative fitness of a resistant strain is below that of the sensitive strain, the resistant strain will only be present at a low mutation–selection level (Equation 6). While it can convert to a fitter, compensated mutant (corresponding to the dashed arrows in Figure 2), it cannot emerge. Population-wide emergence of a resistant strain can only happen if its between-host fitness (*R*_0_) is larger than that of the sensitive strain. If emergence is possible, one can compute approximations for the times at which resistant strain *I* reaches a specified level *L* (Equation 7). In line with epidemiological considerations for gonorrhea [49], we choose *L* as 5% of the total number of infecteds. One finds that the time to emergence not only depends on the rate of introductions *Q,* but also on the relative fitness of the sensitive compared with the resistant strain. While *Q* can either increase or decrease with increasing treatment, the relative fitness of the resistant strains always increases with treatment. Because this latter effect turns out to be the more important one, increases in treatment level directly lead to decreases in the time to emergence, as seen in Figure 4.

**Figure 4:**
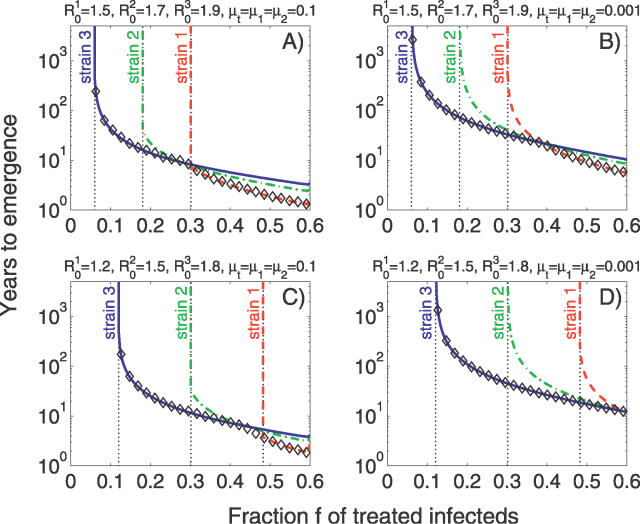
Years until Resistance Emerges—Deterministic Model The black diamonds show the earliest time at which any one of the resistant strains emerges (reaches a level of 5% of total infecteds), obtained from simulations of the full deterministic system (Equation 1). The red dashed, green dash-dotted, and blue solid lines show the analytic approximation (Equation 7) for the time to emergence of the first, second, and third resistant strain. The vertical black lines indicate the level of treatment at which the fitness (*R*_0_) of the respective resistant strain is the same as that of the sensitive. For the top panels, fitness levels of the resistant strains are 75%, 85%, and 95% of the sensitive strain in the absence of treatment, resulting in values for the basic reproductive numbers as indicated. For the bottom panels, fitness levels of the resistant strains are 60%, 75%, and 90% of the sensitive strain. The left panels show results for conversion rates *μ_t_* = *μ*_1_ = *μ*_2_ = 10^−1^, the right panels show results for *μ_t_* = *μ*_1_ = *μ*_2_ = 10^−3^. Other parameter choices are given in Table 1.

### Probability of Emergence for the Stochastic Model

The results shown in Figure 4 are obtained using a deterministic model. Deterministic models provide good approximations when the populations as well as the rate parameters are not too small. In the deterministic model, every resistant strain gets seeded at *t* = 0 at a level depending on the conversion rates and then grows or decays in an exponential fashion, depending on its relative fitness compared with the sensitive strain (Equation 5). However, since the rates of conversion and levels of resistant strains are small, stochasticity can lead to extinction of newly created resistant mutants, until finally one of them takes hold and grows in an essentially deterministic fashion. It is not clear how well the deterministic model can describe the underlying stochastic process, making comparison with a stochastic model necessary. We again consider the endemic scenario. Using a branching process formalism [50–53], we can compute the probability *p* that a single introduction of a resistant strain leads to population-wide resistance emergence (Equation 8), and from that we can estimate the overall probability of emergence (Equation 9). Figure 5 shows P_1_, the probability of emergence within one year, as a function of the level of treatment *f*. We also show results obtained from stochastic simulations, utilizing the well-known Gillespie algorithm [54–56].

**Figure 5:**
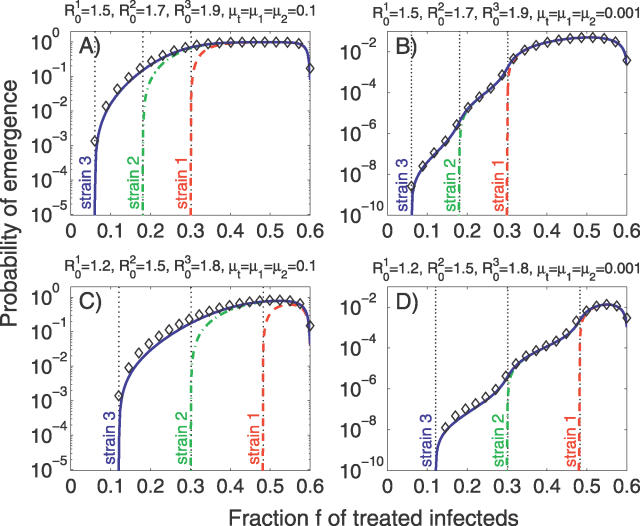
Probability That Resistance Emerges within One Year The black diamonds show the probability that any one of the resistant strains emerges (reaches a level of 5% of total infecteds), obtained from stochastic simulations of the full system (stochastic version of Equation 1). The red dashed, green dash-dotted, and blue solid lines show the analytic result (Equation 9 with *τ* =1). The vertical black lines indicate the level of treatment at which the fitness of the respective resistant strain is the same as that of the sensitive strain. Parameters are chosen as in Figure 4.

### Time to Emergence for the Stochastic Model

We can use the expression obtained for the probability of emergence to estimate the time until emergence occurs. If the probability that a single introduction leads to emergence is *p,* then on average 1/*p* introductions are needed for certain emergence. Because each introduction occurs on average after time 1/*Q,* the time it takes until a process starts to emerge is of order 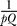 . To get an estimate for the total time it takes for a resistant strain to reach a level *L,* we can add the deterministic time *T_i_* obtained earlier (Equation 7) and find 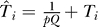 . The result shows that if *pQ* is of order one, the deterministic and stochastic results are expected to give similar results. However, as Figures 3 and 5 show, *pQ* can be significantly less than one, especially for low treatment levels and small conversion probabilities. Therefore, the stochastic component can contribute significantly to the overall time to emergence. Figure 6 shows 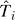 together with results from stochastic simulations. For comparison, we also show the time to emergence obtained earlier from the deterministic model.

**Figure 6:**
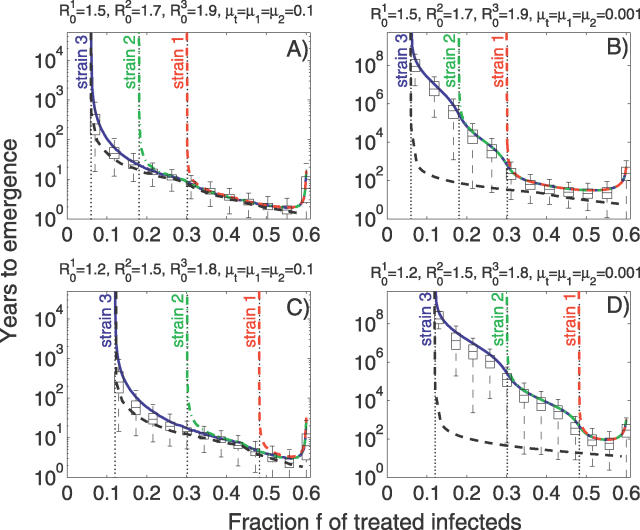
Years until Resistance Emerges—Stochastic Model Boxplots show distribution of times to emergence (resistance at a level of 5% of total infecteds) for 5,000 simulations of the stochastic model. The red dashed, green dash-dotted, and blue solid lines show the analytic approximations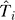 for the time to emergence of the first, second, and third resistant strain. The vertical black lines indicate the level of treatment at which the fitness of the respective resistant strain is the same as that of the sensitive. For comparison, the black dashed line shows the deterministic result (Equation 7). Parameters are chosen as in Figure 4.

### Dependence of Time to Emergence on the Model Parameters

We end our analysis by briefly studying how changes in the number of resistant strains, levels of fitness, and conversion probabilities influence the results. Some examples are shown in Figure 7. Figure 7A confirms the expectation that if more compensatory mutation steps are required to achieve a certain level of fitness, the time to resistance emergence increases. Figure 7B shows how differences in conversion rates and number of compensatory mutation steps influence the time to emergence. It also shows that for small conversion probabilities, small changes in the level of treatment can result in significant changes in the emergence time. Figure 7C shows that for a fixed number of mutation steps, smaller conversion probabilities lead to an increase in time until resistance emerges. Figure 7D shows that even if the number of compensatory steps as well as the product of all conversion probabilities is kept fixed, one obtains varying results for the emergence time, depending on the values of the conversion probabilities.

**Figure 7:**
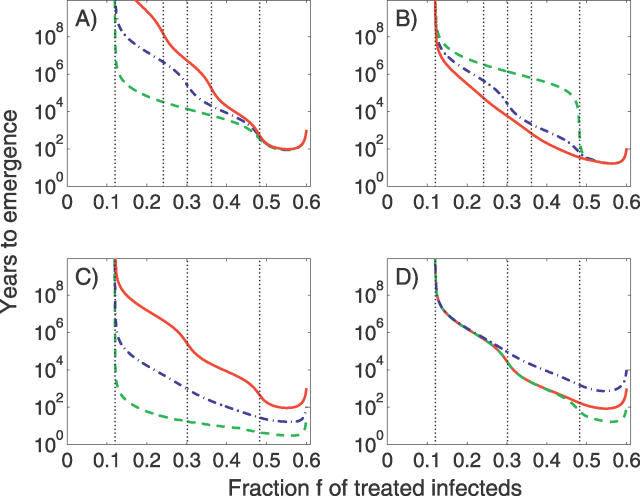
Years until Emergence Occurs as a Function of Treatment (A) All conversion probabilities are *μ_i_* = 10^−3^. The green dashed line shows a situation with two resistant strains with fitness 60% and 90% that of the sensitive strain. The blue dash-dotted line shows three resistant strains with fitness 60%, 75%, and 90%, and the red solid line shows four resistant strains with fitness 60%, 70%, 80%, and 90%. (B) Same number of strains and fitness levels as (A) but the product of all conversion probabilities is kept the same. We choose *μ_t_* = 10^−2^ for all three cases and *μ*_1_ = 10^−6^ for the two-strain scenario (dashed green line), *μ*_1_ = *μ*_2_ = 10^−3^ for the three-strain scenario (dash-dotted blue line), and *μ*_1_ = *μ*_2_ = *μ*_3_ = 10^−2^ for the four-strain scenario (solid red line). (C) Three resistant strains with fitness of 60%, 75%, and 90%. Conversion rates are *μ_i_* = 10^−1^ (dashed green line), *μ_i_* = 10^−2^ (dash-dotted blue line), and *μ_i_* = 10^−3^ (solid red line). (D) Same as (C) but with conversion rates *μ_t_* = 10^−2^, *μ*_1_ = *μ*_2_ = 10^−3^ (dashed green line), *μ_t_* = 10^−4^, *μ*_1_ = *μ*_2_ = 10^−2^ (dash-dotted blue line), and *μ_t_* = *μ*_2_ = 10^−3^, *μ*_1_ = 10^−2^ (solid red line).

## Discussion

Before we discuss in detail the results, we want to stress that while we chose a model and parameters to describe gonorrhea transmission dynamics, the qualitative results we obtained are not limited to gonorrhea and should hold for other diseases as well.

### Implications of Results

While not unexpected, our first and arguably most important finding is that compensatory mutations significantly influence the process of resistance emergence. With the inclusion of compensatory mutations, it is possible that a nonfit resistant strain undergoes further mutations before it goes extinct, until it has regained enough fitness to be able to compete with the sensitive strain (see Figure 2). As an example of this effect, consider the results shown in Figure 5. The red dashed lines show the probability of resistance emergence if no compensatory mutations would occur. For a resistant strain that has 75% fitness compared with the sensitive strain, any treatment level below ≈30% would lead to a zero probability of emergence (top panels). If the fitness were reduced to 60%, treatment of almost 50% of the infecteds would not allow resistance to emerge (bottom panels). However, inclusion of compensatory mutations leads to a more pessimistic outlook. For instance, if the fitness of the most evolved resistant strain is 95%, a treatment level of ≈6% still leads to a nonzero, albeit potentially small, probability of emergence (top panels).

A second important finding is the nonlinear dependence of the probability of and time to emergence on the treatment level. In general, small changes in treatment levels at low *f* lead to much larger changes in the results when compared with changes in *f* at high treatment levels (Figures 5 and 6). The regions of high and low sensitivity to treatment changes depend on the exact nature of the underlying system. For high conversion rates, the curves are relatively smooth, while low conversion rates can result in sharp step-like changes. For instance, if it were known that the dynamics of a specific host–pathogen system is described by the dashed green curve in Figure 7B, one could increase treatment level from ≈20% to ≈45% without significantly reducing the time to resistance emergence. However, even small further increases would lead to a significant drop in the time to emergence. This likely generic feature, which changes in treatment level do not necessarily lead to proportional changes in time to resistance emergence, could have important implications for treatment strategies as well as for the interpretation of epidemiological data.

An additional interesting result is that for treatment levels close to the extinction threshold, it can be beneficial to increase treatment further. Not only might it lead to eradication of the pathogen, but even if eradication does not happen, the low prevalence of infecteds will lead to very few introductions of resistance (Figure 3). If the number of resistance introductions becomes small enough, this effect dominates over the increase in relative fitness of the resistant strains with treatment increase, leading to an overall increase in the average time to emergence (Figure 6). Therefore, while for most treatment levels increased treatment leads to faster resistance emergence, under certain circumstance increasing the treatment level can decrease the probability of resistance emergence.

Our final result concerns the choice of modeling framework. Since the process of compensatory mutations has low numbers and low rates, stochastic effects become important. As Figure 6B and Figure 6D show, results obtained from the deterministic model can differ significantly from the stochastic results. As mentioned earlier, this discrepancy exists because in the stochastic model, introductions of resistance can be followed by extinction, until a resistant pathogen is able to emerge. If the introduction occurs at low frequency, the time until a resistant strain finally starts the emergence process can be potentially large. In contrast to that, the deterministic model seeds all resistant strains immediately, and the emergence process starts without delay. Since compensatory mutations likely play a role in the resistance emergence of most pathogens, we suggest that stochastic models are more appropriate to investigate the resistance emergence process.

### Toward Quantitative Predictions of Resistance Emergence

To quantitatively predict resistance emergence, as well as to design optimal drug-use strategies, one needs detailed knowledge of the parameters and dynamics of the underlying system.

As discussed in the introduction, current lack of data unfortunately limits the quantitative and predictive power of our model. Several steps need to be taken to move toward quantitative results. First and foremost, it is important to obtain good estimates for the parameters involved in resistance emergence. The most straightforward way of doing so would be through epidemiological studies. One would need to identify individuals infected with resistant mutants and determine the clinically relevant routes of compensatory mutation a particular resistant pathogen can follow. Further, one would need to determine the rate at which hosts infected with each mutant arise, and measure the number of secondary infections a host infected with a specific mutant causes. These measurements would directly give parameter estimates for *R*0, the conversion rates *μ_i_,* and the compensatory mutation pathways that are important in the process of emergence. Unfortunately, this approach is as impossible to implement as it is straightforward. It is already difficult to estimate *R*_0_ for pathogens that infect many hosts. Since potentially few hosts are being infected with the different resistant strains, it is pretty much impossible to detect those infections and obtain useful parameter estimates. There might be a better chance to obtain results in controlled transmission experiments in animals. It would likely be possible to investigate the compensatory mutation paths involved in the emergence of resistance. However, measurements for *R*_0_ and the conversion rates would still be difficult to obtain. We think that a more promising approach is to try and obtain the needed parameter estimates by utilizing within-host data and appropriate theoretical models. Measurements of in vivo mutation rates, fitness levels, and pathogen dynamics, combined with appropriate models, might allow estimation of the relative between-host fitness levels for the resistant mutants. Some preliminary work in this direction has been done [41,57], and we are currently in the process of extending some of these results.

Once better and more complete parameter estimates are available, another step toward quantitative predictions can be made by creating more complex epidemiological models. For gonorrhea transmission dynamics, a more realistic model would accurately describe the population and disease life-history by including factors such as gender, sexual activity, sexual orientation, ethnicity, social status, and others [44,45].

Another step toward quantitative predictions will involve more detailed models of the process of resistance emergence. For instance, in our model we assume that treatment does not influence the resistant pathogen. However, in vitro studies of E. coli have shown that the presence of drugs can lead to different compensatory mutations compared with their absence [58]. It is not clear if this is important for in vivo evolution. However, since it is usually not known if a host is infected with a resistant strain or not, potentially resulting in a resistant host receiving treatment, this finding needs to be kept in mind. Further, in our model we assume that a host is always dominated by a single strain. In reality, a host might be infected with a mix of different strains which upon transmission might infect a new host either with one of the transmitted strains or a mixed inoculum, calling for a more detailed model that can incorporate mixed-inoculum effects. We also assumed that conversions happen with a constant probability during the infection and that upon conversion, a host enters a new class, basically starting the infection over. Our model also excludes density-dependent effects, which might become important if the resistant strains become frequent.

As always with biological systems, including every detail would lead to a model that is very complicated and difficult to analyze. Often, simplified models are able to capture the dynamics of complex systems, even on a quantitative level. Without additional studies, it is difficult to predict which biological details will need to be included if one wants to obtain quantitative predictions. However, currently the most serious limitation concerning quantitative results is not the lack of model complexity but the almost complete lack of data for the parameters that govern resistance emergence.

In summary, we obtained important qualitative insights into the effects that compensatory mutations have on the population-wide emergence of drug resistance. Our model used parameters applicable for gonorrhea; however, the results we obtained are rather general. Our finding that the inclusion of compensatory mutations significantly influences the dynamics of resistance emergence has important implications for the design of detailed, predictive models. Especially the fact that changes in the level of treatment can lead to disproportionate changes in the probability of and time to resistance emergence has important public health implications. In future work, it will be necessary to obtain estimates for the currently unknown parameters. This knowledge, combined with more complex and detailed epidemiological models, can ultimately lead to quantitative predictions of how the level of treatment influences emergence of resistance. Such knowledge will be extremely valuable for the rational implementation of treatment strategies.
