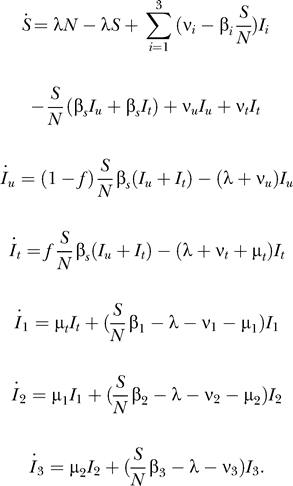# Correction: The Role of Compensatory Mutations in the Emergence of Drug Resistance

**DOI:** 10.1371/journal.pcbi.0030046

**Published:** 2007-02-23

**Authors:** Andreas Handel, Roland R Regoes, Rustom Antia

In *PLoS Computational Biology*, volume 2, issue 10: doi:10.1371/journal.pcbi.0020137

Equation 1 contained typographical errors in the first and last lines. The correct equation is as follows: